# Does Treatment for Depression With Collaborative Care Improve the Glycemic Levels in Diabetic Patients with Depression? A Systematic Review

**DOI:** 10.7759/cureus.10551

**Published:** 2020-09-20

**Authors:** Liliana Diaz Bustamante, Kyrillos N Ghattas, Shahbakht Ilyas, Reham Al-Refai, Reeju Maharjan, Safeera Khan

**Affiliations:** 1 Family Medicine, California Institute of Behavioral Neurosciences and Psychology, Fairfield, USA; 2 Internal Medicine, California Institute of Behavioral Neurosciences and Psychology, Fairfield, USA; 3 Surgery, California Institute of Behavioral Neurosciences and Psychology, Fairfield, USA; 4 Medicine and Surgery, CMH Lahore Medical College and Institute of Dentistry, Lahore, PAK; 5 Pathology, California Institute of Behavioral Neurosciences and Psychology, Fairfield, USA; 6 Neurology, California Institute of Behavioral Neurosciences and Psychology, Fairfield, USA

**Keywords:** diabetes improvement with depression treatment

## Abstract

Diabetes is a chronic disease with a high prevalence in the United States. If not treated adequately, it can have serious complications. Furthermore, when depression affects concomitantly, adherence to treatment can be decreased. Therefore, a cascade of complications may develop, affecting the quality of life and increasing the risk of death.

Depression is underdiagnosed in patients with diabetes, and even if diagnosed, the treatment for both diabetes and depression is not well established in primary care. This study aims to evaluate if treatment for depression with collaborative care can improve glycemic levels and depression treatment response in diabetic patients with depression. As well, we will investigate if treatment with antidepressants will aid in improving glycemic levels. For this systematic review, we followed Preferred Reporting Items for Systematic Reviews and Meta-Analysis (PRISMA) guidelines and used PubMed, PubMed Central, and MEDLINE as database. Keywords: “diabetes improvement with depression treatment’.

For collaborative care intervention, we selected three systematic reviews and meta-analysis. These three studies gave us a total of 1637 patients evaluated for the glycemic outcome and 1793 patients for depression outcomes. For the intervention with antidepressants, we included two articles. One systematic review and meta-analysis that evaluated the effect of selective serotonin reuptake inhibitors (SSRIs) on glycemic levels and the second article involved was a systematic review that assessed the effect of antidepressants on glycemia. A total of 4119 diabetic patients taking antidepressants were evaluated for glucose levels of the outcome. For the collaborative care outcome: two of the three studies showed non-significant improvement of glycemic levels with intervention. However, one study that had a bigger sample size exhibited significant improvement of glycemia with collaborative care. It is necessary to elaborate on new studies to confirm this finding.

For the glycemic outcome with antidepressants: SSRIs improve glycemic levels. This class of antidepressants is the most studied, and it would be interesting to perform trials comparing different classes of antidepressants with a bigger sample size and run for a more extended period. According to our review, collaborative care improves glycemia and depression treatment response. At the same time, it improves the adherence to treatment of both oral hypoglycemic drugs and antidepressants. SSRIs demonstrated to be more effective in glycemic control. The most studied and effective SSRIs are fluoxetine, escitalopram, and citalopram.

## Introduction and background

Diabetes is a common chronic disease that has been increasing over the years. According to the Centers for Disease Control and Prevention’s National Diabetes Statistics Report, 2020, 1.5 million Americans receive the diagnosis of diabetes per year. In 2018 it was estimated that 10.5% of the American population was affected by diabetes. This is equivalent to 34.2 million Americans with this disease [1]. Depression is one of the most common treatable mental health diseases, and it is estimated that around 18 million American adults are affected by it, yearly [2]. Diabetes is accompanied by depression in a percentage range that goes between 11% and 31% [3,4]. This comorbidity can affect the patients not only physically, but also in different aspects such as decreasing their quality of life [5,6], increasing the use of health care system and it's cost [7,8].

Different studies have demonstrated that both diseases together can affect adherence to diabetes treatment [9]. This can be reflected in poor glycemic control [10,11], and consequently, microvascular and macrovascular complications can occur [12]. The worst outcome of this possible cascade of events is mortality, which can also be increased by this disease association [13,14]. Despite this important data, depression is still non diagnosed in 45% of patients with diabetes [15].

Even when depression is diagnosed in diabetic patients, the management of these two comorbidities is not very well established in primary care [16,17]. It is common knowledge that the treatment of depression will cause an improvement of depressive symptoms, but does depression treatment cause an improvement of glycemic levels in a diabetic patient? This question has been the cause of different studies that evaluated the effect of antidepressants and/or collaborative care in glycemic levels, among other outcomes. However, some studies do exhibit a positive effect on glycemic levels, whereas others do not show any significant improvement. 

Due to these mixed results, we conducted a systematic review of prior systematic reviews to determine if the treatment of depression will help in controlling the glycemic levels in patients with both diabetes and depression. We subdivided this study by analyzing the glycemic outcome separately with collaborative care and with the use of antidepressants. We will compare the use of antidepressants with collaborative care to evaluate which approach is the one that provides the best glycemic control. We determined to investigate this topic because we would like to remark on the high prevalence of depression in diabetic patients and its detrimental consequences if not diagnosed or treated properly. We also hope to provide our colleagues and health practitioners in general, with the evidence that will help them decide on which approach is the most adequate for their patients. In this way, we can contribute to improve the quality of life on patients [5,6], decrease complications [10-14], and decrease the health expenses [8].

## Review

Methods

This systematic review was conducted according to Preferred Reporting in Systematic Reviews and Meta-analyses (PRISMA) guidelines.

Search Strategy

We searched for literature from May 25th, 2020, to June 8th, 2020. Our main databases were PubMed, PubMed Central, and MEDLINE. Due to the considerable number of studies involving diabetes and depression, we used specific keywords: “diabetes improvement with depression treatment.” The filters were limited to publication dates within the last ten years and included all article types except for books and documents.

Study Selection

The studies were initially screened by reading the titles and abstracts. Review articles that did not include methods in their abstract were not considered, neither the ones that were irrelevant to our study. Once the abstract seemed adequate, we read the full-text article to analyze the data in more detail. We ruled out studies that did not meet the inclusion criteria. Since all the articles retrieved were systematic reviews and/or conducted a meta-analysis, we checked their quality with the AMSTAR checklist. All the data was collected ethically and legally.

Inclusion Criteria

We included studies that included glycemic levels and depression outcomes after treatment with collaborative care and articles that included the effect of antidepressants on the glycemic level in diabetic patients with depression. The studies chosen were published in the past ten years. We screened Randomized Control Trials (RCTs), systematic reviews, meta-analysis, clinical trials, and reviews. All the included studies focussed on patients that were above 18 years old. We selected both full-text articles and abstracts mentioning the methods section.

Exclusion Criteria

The studies that discussed cognitive behavioral therapy alone or any other psychologic intervention without the integrative perspective and/or multidisciplinary approach were not included. We also excluded the articles without detailing the specific intervention or without measuring glycemic values after the intervention.

Results

Search Results

**Figure 1:**
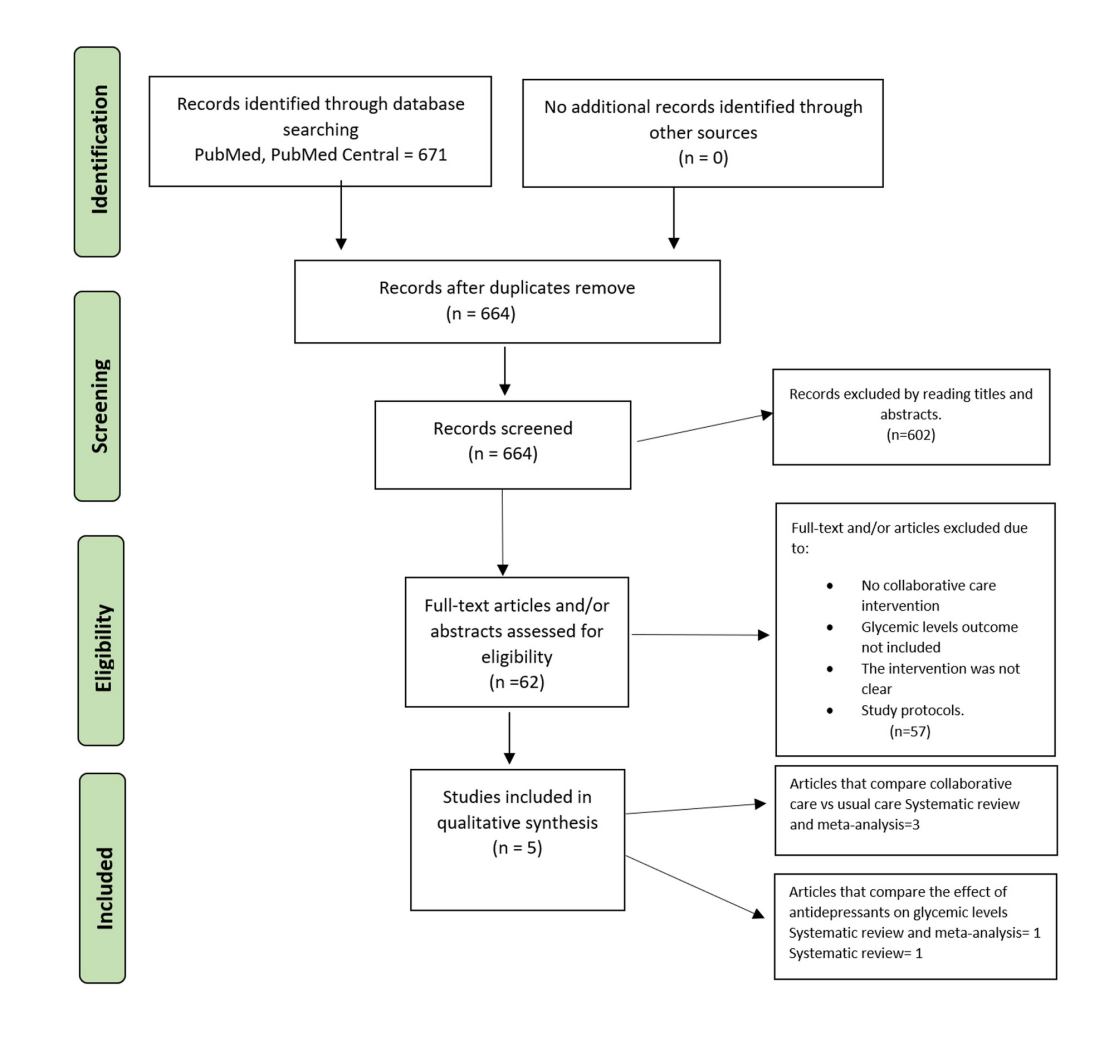
PRISMA diagram and the process of selecting studies. PRISMA: Preferred Reporting in Systematic Reviews and Meta-analyses

The keyword combination, “diabetes improvement with depression treatment,” gave us a total of 671 articles. We read the title and abstracts to perform our first screening and removed 602 studies that were not related to our research question. As well, we excluded seven studies due to five of them being included in systematic reviews, and two of them were repeated. Figure 1 identifies the PRISMA diagram and study selection data.

We pre-selected 57 articles that investigated the effect of depression treatment on glycemic levels. From this point, we selected data according to two interventions. The first one consisted of gathering studies that investigated the glycemic level and depression response after treatment with collaborative care in diabetic patients who also had depression. The second data recollection was based on the intervention that evaluated the glycemic and depression outcome after treatment with collaborative care in patients who had diabetes and depression as comorbidity. Due to this criterion, we excluded studies that treated depression with medications other than antidepressants. At the same time, we screened out the articles that considered depression therapy based on cognitive behavioral therapy, coaching, and any other psychological therapy that did not involve collaborative care or did not have a multidisciplinary approach.

This final screening left us with five articles that were printed in their full version, and since all of them were systematic reviews and/or meta-analysis, we checked for their quality with the AMSTAR checklist. All the studies were written in English.

Study Characteristics

For collaborative care intervention, we selected three articles [18,19,20]. These studies were both systematic reviews and conducted a meta-analysis. All the RCTs included in the article that Watson et al. elaborated [20] were included in either the study by Huang et al. or Atlantis et al. [18,19]. Despite this, we thought that it would be interesting to add it to evaluate the authors’ perspective and point of view since the RCTs were mixed differently in the other two systematic reviews/meta-analyses [18,19]. We were careful to delete the repeated population in our systematic review. These reviews collectively had thirteen studies [21-34].

These three studies gave us a total of 1637 patients evaluated for glycemic levels and 1793 patients for depression outcomes. We summarize these three studies in Table 1.

**Table 1:** Collaborative care: depression and glycemic levels outcome RCT: randomized controlled trial; SOE: strength of evidence; HbA1C: hemoglobin A1c

Author	Title	Year	Study design	Purpose of the study	Intervention	Result/conclusion	Patients studied
Huang et al. [18]	Collaborative care for patients with depression and diabetes mellitus: a systematic review and meta-analysis.	2013	Systematic review and Meta-analysis	Evaluate if collaborative care improves the outcome of depression and diabetes.	Collaborative care vs. usual care	The meta-analysis showed that collaborative care had a significantly increased response to depression and increased treatment adherence to Diabetes and Depression. Reduction in HBA1c levels was not statistically significant in the meta-analysis. However, in the quality study, three RCTs showed improvement of HbA1c levels [26,23,33].	Total of 2238 patients with depression and diabetes. For HbA1c outcome followed at 6 months= 1101. Response to depression treatment followed at 6months= 1118
Watson et al. [20]	Practice-Based Interventions Addressing Concomitant Depression and Chronic Medical Conditions in the Primary Care Setting: A Systematic Review and Meta-Analysis.	2013	Systematic review and Meta-analysis	Evaluate how effective are the interventions for depression or depression and chronic disease.	Collaborative care vs. usual or enhanced care	No difference in glycemic levels between usual care and intervention—conflicting findings with low strength of evidence (SOE). The intervention showed significant improvement in response, symptoms, and remission of depression over usual care. Moderate SOE.	For depression outcome: Total= 930. For HbA1c outcome: Total= 1018
Atlantis et al. [19]	Collaborative care for comorbid depression and diabetes: a systematic review and meta-analysis.	2014	Systematic review and Meta-analysis	Evaluate if depression and glycemic levels can improve with Collaborative care.	Collaborative care vs. usual or enhanced care	Significant improvement in depression and glycemic levels with collaborative care. Depression improvement is not related to glycemic improvement. Both improvements are independent. Studies that included integrated care for diabetes and depression had a better outcome for HbA1c [23,26].	For depression outcome=1895 patients. For HbA1c outcome: 1556.

For the glycemic outcome of diabetic patients treated with antidepressants, we used two articles. One systematic review and meta-analysis evaluated the effect of SSRIs on glycemic levels [21]. The second article we selected is a systematic review that appraised the effect of antidepressants on glycemia [22]. A total of 4119 diabetic patients taking antidepressants were evaluated for glucose levels of the outcome. We summarize the two articles in Table 2.

**Table 2:** Antidepressant's effect on glycemic levels SSRI: selective serotonin reuptake inhibitor; RCT: randomized controlled trial

Author	Title	Year	Study design	Purpose of the study	Interventions studied	Result/conclusion	Articles included	Number of patients
Roopan et al. [22]	Use of antidepressants in patients with depression and comorbid diabetes mellitus: a systematic review	2016	Systematic Review	To find the appropriate antidepressant to be used in diabetic patients with diabetes.	Follow the depression response and glycemic levels after giving antidepressants. Some articles compared antidepressants among them, and in other articles, the antidepressants were compared to placebo.	Most of the studies showed improvement in depression with the use of antidepressants. There were more studies for SSRIs than other groups of antidepressants. SSRIs showed a good response to depression and glycemic levels. The improvement of depression is related to glycemic level improvement. Nevertheless, it is not a cause-effect relationship.	18 studies (17 clinical trials and one case-control).	3695 participants for the glycemic outcome.
Tharmaraja et al. [21]	The Association Between Selective Serotonin Reuptake Inhibitors and Glycemia: A Systematic Review and Meta-Analysis of Randomized Controlled Trials.	2019	Systematic Review and Meta-analysis	To evaluate if SSRIs affect glycemic levels.	SSRIs vs. placebo	SSRIs improved glycemic levels over placebo. Fluoxetine, escitalopram, and citalopram were superior to placebo. Paroxetine was not.	18 RCTs	A total of 835 patients for SSRIs effects on glycemia.

Discussion

We conceived this study to elucidate whether collaborative care treatment for depression can improve depression and the glycemic levels in diabetic patients with depression. In parallel, we also investigated if the use of antidepressants aids in glycemic control in this population.

Collaborative Care Treatment and HBA1c Outcome

Three systematic reviews and meta-analysis matched our search criteria [18,19,20]. One of them is Atlantis et al., which was published in 2014 and included seven RCTs [19]. It followed 1556 patients for HbA1c outcome after treatment of depression with collaborative care. The meta-analysis showed that the intervention improved the glycemic outcome. Interestingly, this is the only meta-analysis that shows significant improvement in the HbA1c levels with intervention. One of the reasons may be that it included new RCTs such as and Bogner et al. and Morgan et al. that were not included in the other two meta-analyses [23,24]. These two studies showed significant improvement in HbA1c levels with intervention. Each of these studies had few differences from the other RCTs that may have influenced the positive results towards intervention. For example, Morgan et al. [24] included in their intervention a follow up with a nurse every three months to assess for risk factors, referrals, depression self-care, planning personal goals, and lifestyle [24]. On the other hand, Bogner et al. offered integrated care involving depression and diabetes care [23].

Atlantis et al. concluded that there is a significant positive effect of collaborative care in HbA1c levels and depression [19]. However, according to their analysis, having a good depression response does not secure or predict a good response in glycemic levels. They believe that the improvement of HbA1c levels may be due to the improvement of self-management.

Watson et al. published in 2013, the second study that we included in our systematic review [20]. It is a huge study that includes twelve studies recruiting a total of 24 RCTs. They aimed to evaluate the effectiveness of the interventions based on one type of care, having as a goal to improve depression or depression and chronic disease. They investigated the outcome of depression in different diseases such as cancer and arthritis, among others. However, we gathered data from the sections that performed intervention and meta-analysis on diabetic patients with depression. As a result, we found that the authors included three RCTs with these characteristics [25,26,27]. In the meta-analyses performed on these studies, the intervention was no different from the usual care. This finding had a low strength of evidence (SOE), according to the authors. One out of three studies found a significant improvement in the HbA1c levels with the intervention [26].

The third study that we included in our review was published in 2013 by Huang et al. [18]. It included eight RCTs [23,26,27,28,29,30,31,32], where five of them studied the outcome of HbA1c after depression treatment with collaborative care and were included in the meta-analysis [26,27,28,29,30]. The results in 1094 patients at the end of the follow-up showed improvement in the glycemic levels but were not statistically significant. Contrarily, the quality assessment showed an HbA1c reduction in three RCTs [23,26,33]. It is interesting to us that the authors did not include in their meta-analysis the study performed by Bogner et al. [23], while it was part of the meta-analysis of Atlantis et al. [19]. We read the methods and results section on both articles, but where not able to find an answer.

This study also included an outcome of the adherence to oral hypoglycemic medication [23,31], which was shown to be significantly improved in the meta-analysis.

The results we found are conflicting since two of them showed no significant improvement of HbA1c levels with collaborative care [18,20], while one showed a substantial reduction in glycemic levels with intervention. We believe that even though the three studies have good quality, some details can help us conclude whether collaborative care aids in improving glycemic levels. We compared these three studies in Table 3.

**Table 3:** Comparison of the three systematic reviews and meta-analysis regarding HbA1c outcome RCT: Randomized controlled trial

Characteristics	Huang et al. [18]	Watson et al. [20]	Atlantis et al. [19]
Number of RCTs included	8	3 (counting specific RCTs for our purpose)	7
Year of Publication	2013	2013	2014
Population studied	1094	1018 (Total number calculated by us)	1556

According to this comparison, Atlantis at al. have a more significant number of patients studied [19]. It is a more recent study and has included new RCTs. It shows encouraging results, and we believe that collaborative care does help in improving glycemic levels. Nevertheless, it is not precisely the finding that we expected, but it gives us a new perspective. We formulated our question, thinking about the outcome of glycemic levels with depression treatment. However, the RCTs with significant results involved more than depression treatment. For example, Morgan et al. assessed risk factors, lifestyle, and personal goals [24].

Similarly, Katon et al. had as an outcome a treat-to-target approach, provided integrated care for diabetes and depression, had a maintenance plan, and stimulated self-care and exercise. As well, we found it interesting that it included motivational problem solving [26]. Another study that integrated care for depression and diabetes was Bogner et al. [23]. Seeing these interesting additions in the studies, we encourage our colleagues to run new RCTs, including these crucial interventions as a part of collaborative care, and to have HbA1c as a primary outcome to follow up. It would be intriguing to evaluate if these new approaches will significantly help in the improvement of glycemic levels.

We can conclude that collaborative care treatment for depression does not improve glycemic levels directly. However, if we add integrated care for diabetes, including managing risk factors, stimulating a healthy lifestyle, and having a maintenance plan, we may obtain promising results.

Limitations:Most of all, the RCTs were performed in the United States. It may not be reproducible in countries with fewer resources as a limited number of health care practitioners per population or no multidisciplinary approach.

Since we limited our search criteria to the past ten years, we may be missing important contributions. As well, we may have missed important contributions by not searching in more than three databases. 

Collaborative Care Treatment for Depression and Depression Outcome

We analyzed three systematic reviews and meta-analysis [18,19,20]. As we mentioned before, the study by Huang et al. [18], included eight RCT’s on their meta-analysis [23,26, 27,28, 29, 30, 31, 32], and four of them were used to analyze the depression outcome [26,28,29,32]. The results in 1096 patients showed that collaborative care does help improving response to depression treatment at six and twelve months, and the end of the follow-up. Also, the analysis for adherence to antidepressant treatment showed a significant increase in adherence to this medication.

Watson et al. [20], included three studies in the meta-analysis, that followed the reduction of depression in diabetic patients with collaborative care [25,26,27]. All the studies showed significant improvement of depression with collaborative care at six and twelve months with a moderate strength of evidence (SOE).

Lastly, Atlantis et al. [19], included seven RCTs in their meta-analysis to assess the outcome of depression with the intervention [23, 24,25, 26, 27,28,30]. It showed that depression improved significantly with collaborative care in 1895, followed by patients.

The three studies conclude that collaborative care is effective in improving depression outcomes. And seeing the quality of the studies, and the constant positive results, we are sure that our question is solved and encourage colleagues overall in primary care to start this type of care for the benefit of the patients since it is efficient. Now the next question would be to investigate if this study is cost-effective and if it is easy to implement in primary care without many resources.

Limitations: The limitations of this study are that most of the studies were developed in the United States, and it may not be reproducible in other countries or realities. For this reason, we encourage investigators from other areas to run trials about implementing collaborative care, considering their reality and resources. We also may have missed relevant studies by limiting our search criteria to ten years from publication.

Treatment with Antidepressants, Glycemic Outcome

It is clear that antidepressants play an important role in depression treatment; for this reason, we mainly focus on glycemic results. To evaluate the antidepressants’ effect on the glycemic levels, we selected two studies. One of them is a systematic review and meta-analysis published in 2019 by Tharmaraja et al. This study aimed to evaluate if SSRIs can alter glycemic levels [21]. The team included 16 RCTs with a total of 835 participants divided into subgroups (depressed, non-depressed, diabetic, and non-diabetic) to analyze the effects of SSRIs on different populations. Ten studies investigated glycemic outcomes in diabetic patients with 424 participants, and five articles followed the glycemic outcome on depressed and diabetic patients conformed by 247 patients. The statistics focused on general and diabetic populations.

In general, SSRIs improved glycemic levels over placebo. In the subgroup of 424 diabetic patients, this improvement was statistically significant. Only five RCTs included both diabetic and depressed patients, but we do not have statistical data for this specific group. Among the SSRIs, citalopram, escitalopram and fluoxetine were superior to placebo, but paroxetine was not. The authors believe that the improvement of glycemia may be due to an increase in insulin release with SSRIs treatment. In the case of depressed patients, it may be due to better self-care, adherence to treatment, and more activity. The authors recommend further investigation of these areas.

The second study included is a systematic review published in 2016 by Roopan et al. [22]. This study looked for adequate antidepressant in patients with depression and concomitant diabetes. It included eighteen articles with different levels of evidence and studied a total of 3695 depressed patients with diabetes. Most of the studies in this systematic review investigated SSRIs, which showed improvement of glycemia. Paile-Hyvarinen et al. noticed that patients taking paroxetine had an increase in sex hormone binding globulin, being an indication of better insulin sensitivity [34]. Despite the findings, the authors did not incline towards a certain type of antidepressants and recommended to run bigger sample RCTs with more detailed attention to glycemic levels.

Roopan et al. has a big sample size, included eighteen articles, and all the participants had depression and diabetes with HbA1c as an outcome [22]. However, we agree with the authors in the sense that there is no possibility to conclude, due to some articles having a high risk of bias for not being randomized and/or blinded, have mixed results, among others.

Tharmaraja et al. showed that SSRIs improved glycemia in diabetic patients. We incline towards this study due to its high level of evidence and good sample size [21]. Even though SSRIs showed a positive result, it is important to elaborate trials for a longer period and with a bigger sample size to confirm their effectivity. As well, it will be relevant to run tests that compare different groups of antidepressants as most of the studies have investigated SSRIs.

Limitations: We may be missing important data by filtering away publications older than ten years.

## Conclusions

According to our review, collaborative care improves glycemia and depression treatment response. Significantly, it improves the adherence to treatment of both oral hypoglycemic drugs and antidepressants. We found that some other good interventions may be the assessment for risk factors, giving resources such as problem-solving skills, setting goals, and promoting self-care for depression to the participants.

Regarding the antidepressants, SSRIs have demonstrated to be more effective in glycemic control. The most studied and effective SSRIs are fluoxetine, escitalopram, and citalopram. Paroxetine was not superior to placebo. We cannot conclude that antidepressants alone are superior to collaborative care or vice versa. Nevertheless, we think that a reasonable approach would be to provide collaborative care for both depression and diabetes with SSRIs treatment. After analyzing the selected studies, we think that collaborative care is an excellent approach in patients with comorbidities such as depression and diabetes. However, we need more studies with a large sample size that evaluate the cost-effectiveness and the implementation process for this type of care. 
